# Ancient Egypt and today: enough scourges to go around.

**DOI:** 10.3201/eid0204.960418

**Published:** 1996

**Authors:** D G Colley


5. World Health Organization. Interim proposal for a WHO staging system for HIV infection and diseases. Wkly Epidemiol Rec 1990;65:221-4.

### Ancient Egypt and Today: Enough Scourges to Go Around

**To the Editor:** In a recent letter (1), Ablin conjectures that translation of the hieroglyphic symbol for *AAA* in many ancient Egyptian papyri (Ebers, Berlin, Hearst, London, and Kahum), may be suggesting the existence of human immunodeficiency virus (HIV) or its prototype during the time of the pharaohs. While hieroglyphic interpretations remain challenging, the symbol cited in his letter has most commonly been translated as hematuria (2-4) and has most often been related to schistosomiasis haematobia. This infection, caused by the helminth *Schistosoma haematobium*, has been shown to have occurred in Egypt from early pharaonic times (3200 B.C.), by the demonstration of schistosome eggs (5) and circulating schistosome antigens (6,7) in mummies. Remedies for hematuria were recorded in papyri from many centuries (9 in Hearst, 11 in Berlin, 20 in Ebers), perhaps implying that the condition was serious and widespread. In giving one of the remedies in the Ebers papyrus (circa 1500 B.C.), the text actually mentions worms in the body (although it seems to state that the worms are caused by *AAA* disease, perhaps inverting the true order of causality). In the Hearst papyrus one of the remedies cited for hematuria is antimony disulfide. Until only 25 years ago, antimonial compounds were the most effective drugs for schistosomiasis chemotherapy.

It seems likely that, over a period of many centuries in ancient Egypt, *AAA* disease was a widespread condition of sufficient severity to require medical attention. I concur with many others in proposing that the translation of *AAA* disease is hematuria, and that the relationship drawn between *AAA* and worms in the body, antimonial-based remedies, and the knowledge that *S. haematobium* infections were widely present at that time provide strong evidence that *AAA* disease refers to schistosomiasis haematobia.

Schistosomiasis is still with us. In fact, through dispersions of both human populations and specific fresh-water snails (the intermediate hosts for schistosomes), this disease now infects some 200 million persons and is responsible for an estimated 800,000 deaths per year (8). While clearly ancient, schistosomiasis can emerge as a new infectious disease in a given location under certain man-made changes in environmental conditions and economic- or war-related migrations of people. For example, in the Senegal River basin, estuarine dams, irrigation systems, and an influx of people to work irrigation-intense crops led, over a period of only 3 years, to an increased prevalence of *S. mansoni* infection from 0% to >95% of the population of >50,000 (9). Even in modern-day Egypt, such interventions as the Aswan High Dam have significantly altered patterns of schistosomiasis (2,10). The Ministry of Health and Population of Egypt and the U.S. Agency for International Development are addressing this ancient scourge through the Schistosomiasis Research Project, a national schistosomiasis research and control program that attacks the disease with available tools, while it presses forward with research on much needed new tools, such as vaccines.

**Daniel G. Colley**
Centers for Disease Control and Prevention,
Atlanta, Georgia, USA

### References

1. Ablin RJ. AIDS: déjà vu in ancient Egypt? Emerging Infectious Diseases 1996;2:242.
2. Abdel-Wahab MF. Schistosomiasis in Egypt. Boca Raton: CRC Press, Inc., 1982.
3. Farooq M. Historical development. In: Ansari N, editor. Epidemiology and control of schistosomiasis (bilharziasis). Baltimore: University Park Press, 1973:1-16.
4. Kolata G. Avoiding the schistosome's tricks. Science 1985;227:285-7.
5. Ruffer M. Note on the presence of *Bilharzia haematobium* in Egyptian mummies of the twentieth dynasty (1250-1000 B.C.). Br Med J 1910;1:16.
6. Deelder AM, Miller RL, de Jonge N, Krijger FW. Detection of antigen in mummies. Lancet 1990;335:724-5.

7. Miller RL, Armelagos GJ, Ikram S, de Jonge N, Krijger FW, Deelder AM. Paleoepidemiology of *Schistosoma* infection in mummies. Br Med J 1992;304:555-6.
8. Capron A, Dessaint JP, Capron M, Ouma JH, Butterworth AE. Immunity to schistosomes: Progress toward a vaccine. Science 1987;238:1065-72.
9. Gryseels B, Stelma FF, Talla I, van Dam GJ, Polman K, Sow S, et al. Epidemiology, immunology and chemotherapy of *Schistosoma mansoni* infections in a recently exposed community in Senegal. Trop Geogr Med 1994;46:209-19.
10. El Alamy MA, Cline BL. Prevalence and intensity of *Schistosoma haematobium* and *S. mansoni* infection in Qalyub, Egypt. Am J Trop Med Hyg 1977;26:470-2.

### AIDS and *ĀĀĀ* in Egypt?

**To the Editor:** A recent letter concerning Egyptian hieroglyphs on the disease *ĀĀĀ* asks if this disease could be AIDS or an HIV-associated condition prevalent in Egypt during the time of the pharaohs (1). We believe this possibility is highly unlikely. Aside from conflicts with current thought on the origin and evolution of lentiviruses, there is a problem of linguistic interpretation. The initial hieroglyph in the series of hieroglyphs comprising the word *ĀĀĀ*, a picture of a discharging phallus, is a "determinative," indicating the class or category to which the word belongs. Although scholars once took this determinative to indicate a phallic connection with disease, even suggesting that *ĀĀĀ* meant hematuria, consistent with schistosomiasis (2,3), it was later proposed that the determinative meant semen or poison, reflecting the Egyptian concept that diseases may be transmitted by an evil spirit in the form of an incubus, impregnating a victim with poisonous semen. This interpretation is now generally accepted (4,5). The phallus-with-discharge thus came to indicate a deadly disease, and *ĀĀĀ* a poisonous disease-causing substance introduced into the body by magic. The word *ĀĀĀ* is used elsewhere in the Egyptian medical papyri in other contexts, such as "*ĀĀĀ* of the heart" and "*ĀĀĀ* of the belly and heart," and is not known to have been used in connection with the bladder or genitalia. While the determinative meaning may not be absolutely established, it is clear from its usage in other contexts that the phallus-with-discharge determinative can indicate fatal or serious illness. The notion that the phallus-with-discharge determinative refers to sexually transmitted disease is not consistent with its usage. To further argue that *ĀĀĀ* represents AIDS or HIV disease is not justified by the linguistic evidence. Without further archaeologic or inscriptional evidence, we would doubt that HIV circulated in ancient Egypt.

**Robert J. Littman and David M. Morens**
University of Hawaii, Honolulu, Hawaii

### References

1. Ablin RJ. Déjà vu in ancient Egypt? Emerging Infectious Diseases 1996;2:242.
2. Ebbell B. The Papyrus Ebers. London: Oxford University Press, 1937.
3. Jonckheere F. Une Maladie Égyptienne, l'Hématurie Parasitaire. Bruxelles: Fondation Égyptologique Reine Élizabeth, 1944.
4. von Deines H, Westendorf W. Wörterbuch der medizinischen Texte. Erste Hälfte. Berlin: Akademie-Verlag, 1961;7(Part 1):129.
5. Nunn JF. Chapter 3. Concepts of anatomy, physiology and pathology. Chapter 4. The pattern of disease. Chapter 5. Magic and religion in medicine. In: Nunn JF: Ancient Egyptian Medicine. London: British Museum Press, 1966:42-63,64-95,96-112.

